# Curcumin-Mediated Sono-Photodynamic Treatment Inactivates *Listeria monocytogenes* via ROS-Induced Physical Disruption and Oxidative Damage

**DOI:** 10.3390/foods11060808

**Published:** 2022-03-11

**Authors:** Jiawen Zhang, Peiying Zheng, Jing Li, Yijing Yang, Shaoxiao Zeng, Jianqing Qiu, Shaoling Lin

**Affiliations:** 1 College of Food Science, Fujian Agriculture and Forestry University, Fuzhou 350002, China; jwgazhang@163.com (J.Z.); zhengpy19990520@163.com (P.Z.); l-eliauk@foxmail.com (J.L.); clemf10@163.com (Y.Y.); zsxfst@163.com (S.Z.); 2 Key Laboratory of Marine Biotechnology of Fujian Province, Institute of Oceanology, Fujian Agriculture and Forestry University, Fuzhou 350002, China; 3 College of Food and Bioengineering, Fujian Polytechnic Normal University, Fuqing 350300, China; qjqqiu@163.com

**Keywords:** sono-photodynamic sterilization, curcumin, *Listeria monocytogenes*, reactive oxygen species

## Abstract

Sono-photodynamic sterilization technology (SPDT) has become a promising non-thermal food sterilization technique because of its high penetrating power and outstanding microbicidal effects. In this study, *Listeria monocytogenes* (*LMO*) was effectively inactivated using curcumin as the sono-photosensitizer activated by ultrasound and blue LED light. The SPDT treatment at optimized conditions yielded a 4-log reduction in *LMO* CFU. The reactive oxygen species (ROS) production in *LMO* upon SPDT treatment was subsequently investigated. The results demonstrated SPDT treatment-induced excessive ROS generation led to bacterial cell deformation and membrane rupture, as revealed by the scanning electron microscope (SEM) and cytoplasmic material leakage. Moreover, agarose gel electrophoresis and SDS-PAGE further revealed that SPDT also triggered bacterial genomic DNA cleavage and protein degradation in *LMO*, thus inducing bacterial apoptosis-like events, such as membrane depolarization.

## 1. Introduction

Pathogenic bacterial contamination is the most significant cause of food safety issues and the most widespread public health concern worldwide [1]. *Listeria monocytogenes* (*LMO*), together with *Escherichia coli*, *Staphylococcus aureus*, and *Salmonella enterica*, are considered the most important bacterial pathogens commonly implicated in food-borne illnesses [2]. *LMO* has been commonly detected in a variety of foods including frozen meat, aquatic products, and dairy ingredients [3]. Moreover, listeriosis caused by *LMO* infection can be particularly dangerous or even fatal for immune-compromised people. For instance, listeriosis is considered extremely dangerous for pregnant women and their newborn babies, resulting in miscarriage, stillbirth or even death [4,5]. Additionally, *LMO* is highly pathogenic due to its secretion of a variety of virulence factors (internalin, phospholipases, hemolysin, and virulence proteins) [6,7,8,9,10,11,12]. Therefore, exploring sanitizing methods against *LMO* has drawn great attention from scholars.

Traditional thermal sterilization techniques transmit large amounts of heat energy to the foodstuff through heat conduction, thereby inactivating microorganisms [13]. Currently, these thermal sterilization techniques have been widely adopted in the food industry [14]. However, thermal sterilization often severely affects the flavor, color and nutrient content of foods [15]. With the continuous development of non-conventional sterilization techniques, a number of non-thermal sterilization technologies, such as ultra-high-pressure, pulsed light, ultrasonic, ultraviolet, and sono-photodynamic treatment (SPDT) have gained significant attention [16]. Particularly, SPDT was effectively applied in inhibiting periopathogenic bacteria with numerous advantages, such as ease of operation, low cost, good penetrating power and safety [17].

Similar to the widely-adopted antimicrobial photodynamic inactivation (aPDI), in which photosensitizer absorbs light energy and catalyzes the formation of ROS, SPDT relies on the sono-photosensitizer activated by light and ultrasound simultaneously to generate ROS [18,19]. Notably, SPDT offers numerous advantages over aPDI. For instance, light often has limited penetration depth, therefore, aPDT is thus only suitable for sterilization of food surfaces and some liquid clarified beverages but not opaque liquids and solid foods. In contrast, ultrasound used in the sono-dynamic technology has strong penetrating power [20] and thus can achieve good sterilization effects on opaque liquids and the interior of solid foods. Additionally, the cavitation of ultrasound also contributes to the microbicidal activity of SPDT [20]. Indeed, Niavarzi et al. [21] have compared the killing effects of methylene blue-mediated aPDI and SPDT against *Enterococcus faecalis* biofilms and found SPDT yields more significant decreases in the survival of *faecalis*. Drantantiyas et al. [22] also reported that sono-photodynamic sterilization techniques were more effective in inhibiting *S. aureus* than sono-dynamic and photodynamic bactericidal techniques alone.

However, searching for a suitable sono-photosensitizer is always a challenge for commercial applications of SPDT in the food industry. Unlike chemically synthesized sono-photosensitizers, curcumin is a naturally-derived polyphenol approved by the WHO and FDA as food additive [23,24]. Therefore, several studies were conducted to explore the potential of curcumin-based SPDT for killing food-borne bacteria and food preservation [25]. For instance, Fernanda et al. [26] demonstrated curcumin-mediated SPDT could achieve a reduction of 3.48-log of *S. aureus* with bacterial biofilm disruption. Similarly, Maryam et al. [27] showed curcumin-decorated nanophytosomes-mediated SPDT could effectively kill *Aggregatibacter actinomycetemcomitans* by more than 10-log reduction of CFU and significantly decrease the bacterial metabolic activity. Bhavya et al. [28] also reported the killing effects of curcumin-mediated SPDT on *E. coli* and *S. aureus* in orange juice; while SPDT was also found to inhibit the growth of the spoilage microorganisms (*Psychrobacter* and *Brochothrix*) in shrimp surimi [29]. However, to the best of our knowledge, the bactericidal activity of curcumin-based SPDT on *LMO*, as well as its underlying mechanisms, have not been reported. Therefore, in the current study, the killing effects of SPDT against *LMO*, along with possible mechanisms of action, were explored. Particularly, the excessive ROS generated during SPDT treatment, as well as its disruption activities on the bacterial membrane, were determined, followed by the assessment of ROS-induced oxidative damage to bacterial DNA and proteins.

## 2. Materials and Methods

### 2.1. Preparation of Bacterial Suspension and Curcumin Solution

*LMO* was obtained from the China Microbial Strain Conservation Centre (CMCC, Beijing, China) and stored at −80 °C. A single colony was transferred to TSB-YE broth and cultured at 37 ± 1 °C. The bacteria were harvested at logarithmic growth stage by centrifugation at 5000× *g* for 5 min at 4 °C. Harvested *LMO* pellets were washed with sterilized PBS solution (pH 7.4) and resuspended in PBS at OD_600_ = 0.5 (≈10^9^ CFU/mL). Curcumin was purchased from MCE China (Shanghai, China). To prepare the curcumin stock solution (10 mM), 185 mg of curcumin was dissolved in 50 mL of ethanol solution and stirred for 30 min on a magnetic stirrer. The curcumin stock solution was stored at 4 °C, and further diluted using PBS before experiment. Since the maximum concentration of curcumin working solution in the current study was 70 μM, the ethanol concentration in all curcumin working solutions was below 0.7%, which has little impact on the viability of bacteria [30].

### 2.2. Sono-Photodynamic Sterilization Treatment

**Figure 1:**
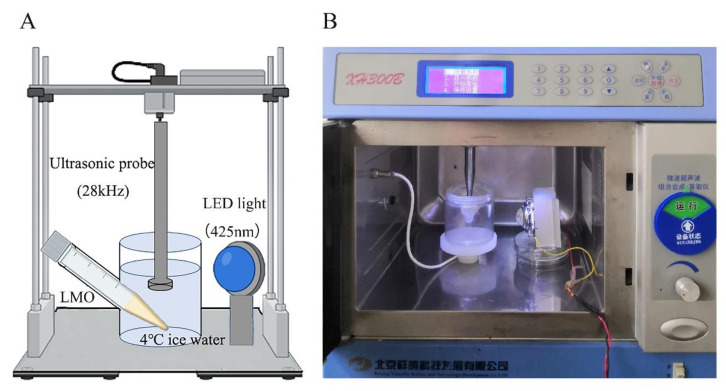
The schematic diagram (**A**) and apparatus (**B**) of sono-photodynamic treatment for inactivating food-borne bacteria *Listeria monocytogenes*.

For the sono-photodynamic sterilization treatment, curcumin solution was added into the bacterial suspension and incubated in the dark for 30 min. Then the bacterial samples were exposed to ultrasound treatment (XH300E, XiangHu Technologies, Beijing, China) and blue LED illumination (M425L, Zolix Instruments Co., Ltd., Beijing, China). The distance between sample and ultrasonic probe and LED bulb was 2 and 5 cm, respectively (as illustrated in Figure 1). Thereafter, the bacterial viability was assessed with CFU counting assay using PCA plates. In brief, ten-fold serial dilutions of *LMO* samples were prepared, and 200 µL of each dilution was incubated on the PCA plate at 37 °C for 24 h. The CFUs were calculated by multiplying the numbers of colonies counted on the plates by the dilution ratio.

### 2.3. Detection of Reactive Oxygen Species (ROS)

The measurement of ROS was performed using DCFH-DA staining method as described by Akhtar et al. [19]. 2,7-dichlorodihydrofluorescein diacetate (DCFH-DA) was used as a fluorescent probe to detect the ROS content [31]. In brief, 500 μL of bacterial solution in each group (~10^9^ CFU/mL before treatments) was mixed with 25 μL of DCFH-DA solution (10 μmol/L) and incubated at 37 °C for 30 min and then the ROS was determined by fluorescence spectrophotometers (RF-5301PC, Shimadzu, Kyoto, Japan). The excitation wavelength (λex), emission wavelength (λem) and slit width were set as λex = 490 nm, λem = 520 nm and 5 nm, respectively.

### 2.4. Scanning Electron Microscopy (SEM)

SEM images were captured according to the method of Lai et al. [23]. In brief, the bacterial solutions (5 mL, at ~10^9^ CFU/mL before treatments) were centrifuged at 5000× *g* for 5 min at 4 °C to harvest the bacteria, which were then loaded on the coverslip and fixed with glutaraldehyde at a concentration of 2.5% for overnight, and then rinsed three times with PBS. The bacteria were fixed with 1% osmium tetroxide for 6 h, followed by stepwise dehydration with 25%, 50%, 75% and 95% ethanol. After drying in a CO_2_ desiccator, the bacterial samples were coated by gold spraying. Finally, the cell structure was observed on a high-resolution field emission scanning electron microscope (SIGMA, Carl Zeiss, Rödermark, Germany).

### 2.5. Determination of Cytoplasmic Material Leakage

*LMO* (at ~10^9^ CFU/mL) was treated as described above. Then the cytoplasmic material leakage was measured according to the method described in the previous studies [23,32]. In brief, 500 μL of bacterial suspension in each group was passed through a 0.22 μm pore size membrane. The OD_260_ and OD_280_ of the filtrates were measured using an ultra-micro UV-Vis spectrophotometer (ND2000C, Thermo Fisher, Waltham, MA, USA).

### 2.6. DNA Agarose Gel Electrophoresis

The bacterial genomic DNA was extracted according to the manufacturer’s protocol (Omega Bio-tek, Norcross, GA, USA). The extracted genomic DNA was mixed with 6 × DNA Loading Buffer and separated in 1% agarose gel. The electrophoresis apparatus parameters were set at 100 V for 30 min. The gel was visualized using gel imaging system (ChemiDoc MP, Bio-Rad, Hercules, CA, USA) after electrophoresis [23].

### 2.7. Polyacrylamide Gel Electrophoresis (SDS-PAGE)

In brief, the bacterial pellet was resuspended in protein lysis buffer (2×). Upon complete lysis of the bacteria, the lysates were mixed with same volume of Protein Loading Dye (2×) and then heated at 100 °C for 5 min. Samples were centrifuged at 10,000× *g* for 30 s and the supernatant was loaded into SDS polyacrylamide gel for electrophoresis. After electrophoresis, the gels were stained with Coomassie Brilliant Blue Dye (P0017, Beyotime, Shanghai, China) and the images were observed using gel imaging system (ChemiDoc MP, Bio-Rad, Hercules, CA, USA).

### 2.8. Annexin V-FITC/PI Staining Assay

The bacterial apoptosis-like event membrane depolarization was evaluated using the Annexin V-FITC Apoptosis Detection Kit (C1062M, Beyotime, Shanghai, China) following the manufacturer’s instructions. The bacterial pellets were harvested by centrifugation, resuspended in PBS and stained with Annexin V-FITC and propidium iodide at 25 °C for 15 min in the dark, followed by flow cytometric detection using Beckman Coulter CytoFLEX (Indianapolis, IN, USA).

### 2.9. Data Statistics and Analysis

All experimental data were obtained from at least three replicates. Data were expressed as mean ± SD. SPSS software (version 24.0) was used for performing statistical analysis and significant differences between groups were determined by one-way analysis of variance (ANOVA) with Duncan’s multiple comparisons. The significance level was 0.05.

## 3. Results

### 3.1. SPDT Showed Effective Bactericidal Activity against LMO

**Figure 2:**
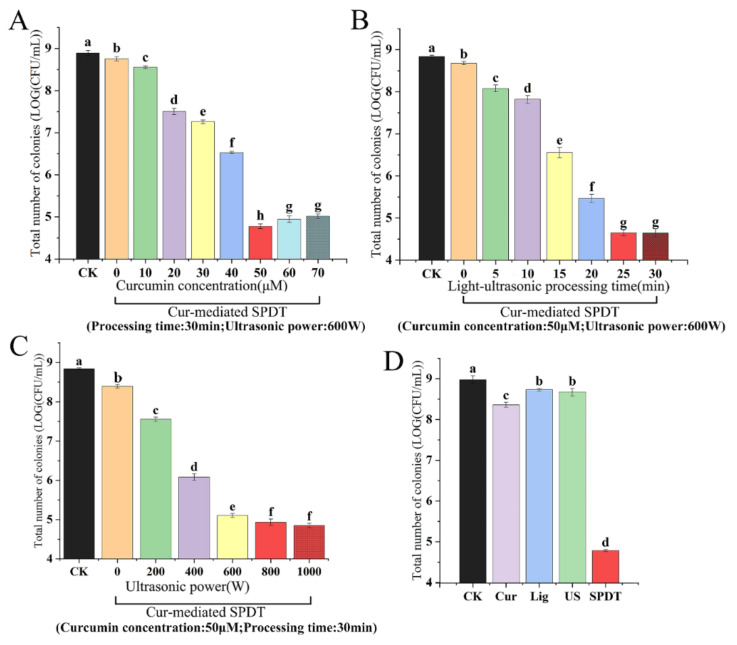
The optimization of curcumin concentration (**A**), light-ultrasonic processing time (**B**) and ultrasonic power (**C**) for the bactericidal activity of curcumin-mediated sono-photodynamic treatment against *Listeria monocytogenes*; (**D**) The bactericidal effects of curcumin only (50 μM), light-illumination only (25 min), ultrasonic treatment only (800 W) and curcumin-mediated SPDT against *LMO*. CK: Control group without treatment; Cur: Curcumin-treated group; Lig: Light-illuminated group; US: Ultrasound-treated group; SPDT: Curcumin-mediated sono-photodynamic treated group. The significant differences among samples were denoted by various lowercase letters (*p* < 0.05).

As shown in Figure 2, the increase in the concentration of curcumin (ranging from 10–50 μmol/L) led to the enhanced killing effects of SPDT against *LMO*. A 4.12-log CFU/mL decrease was observed with curcumin at a concentration of 50 μmol/L (Figure 2A). However, a further increase in the curcumin concentration only enhanced the antibacterial efficacy of SPDT slightly. Notably, increasing the curcumin concentration beyond 60 μM even resulted in decreased killing effects. This phenomenon may be due to the fact that a higher curcumin concentration led to the increase in opacity of the solution, which may hinder the penetration of the lights. Similarly, the number of *LMO* colonies gradually decreased with the increasing duration of the sono-photodynamic treatment. The number of colonies decreased by 4.19-logs when the processing time was 25 min; while the number of *LMO* colonies further decreased slightly when the treatment time was expanded to 30 min (Figure 2B). In addition, ultrasonic power was also identified as an important factor affecting bactericidal activity. The killing effects of SPDT increased rapidly with ultrasonic power rising to 600 W. A 3.91-log decrease in CFU/mL was achieved when the ultrasonic power reached 800 W (Figure 2C). Moreover, the bactericidal effects of negative controls (curcumin only, light-illumination only, and ultrasonic treatment only with their optimized parameters) were next explored. The results also supported that only curcumin-mediated SPDT achieved desirable bactericidal effects against *LMO* (Figure 2D and Figure S1).

### 3.2. SPDT Generated Significant Intracellular ROS in LMO

**Figure 3:**
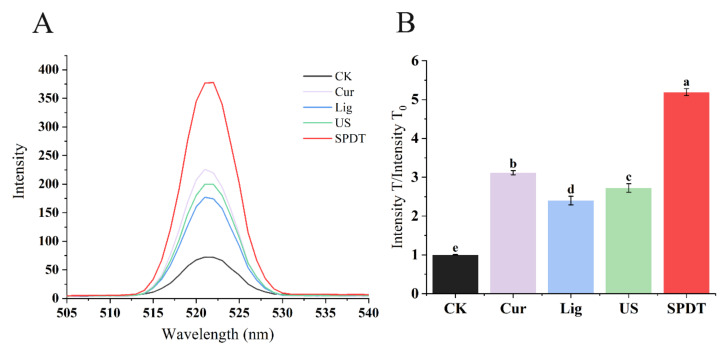
Generation of reactive oxygen species (ROS) in *Listeria monocytogenes* cells subjected to different treatments. (**A**) Fluorescence intensity of *Listeria monocytogenes* in different treatment groups. (**B**) The ratio of the fluorescence intensity of each treatment group to CK group. CK: Control group without treatment; Cur: Curcumin-treated group; Lig: Light-illuminated group; US: Ultrasound-treated group; SPDT: Curcumin-mediated sono-photodynamic treated group. Different lowercase letters above the columns indicate the significant difference (*p* < 0.05).

As shown in Figure 3, the fluorescence in the control groups (curcumin-treated only, light-illuminated only, ultrasonicated only) showed increases in fluorescence of 2~3-fold compared to the untreated bacteria, while the SPDT-treatment was found to augment the fluorescence intensity by >5 folds in the treated bacteria compared with untreated bacteria, indicating significantly more ROS generation occurred in *LMO* upon SPDT treatment (Figure 3).

### 3.3. SPDT Altered Morphology of LMO

**Figure 4:**
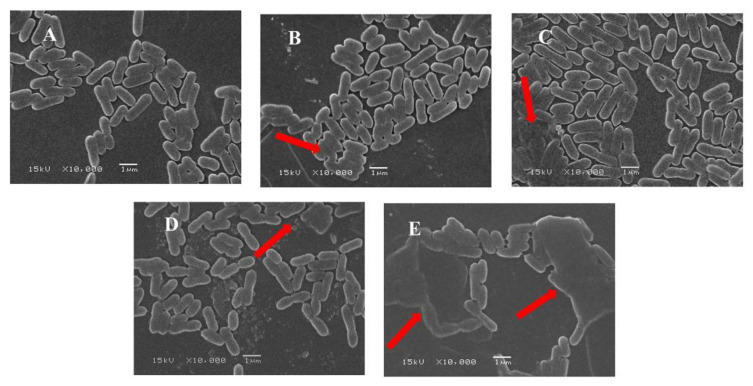
SEM images of *Listeria monocytogenes.* (**A**) Control group without treatment; (**B**) Curcumin-treated group; (**C**) Light-illuminated group; (**D**) Ultrasound-treated group; (**E**) SPDT: Curcumin-mediated sono-photodynamic treated group.

As shown in Figure 4A, the untreated *LMO* cells showed the normal shape of bacteria surrounded by intact cell membranes without the obvious release of intracellular components. A fraction of *LMO* with curcumin treatment or blue-light illumination or ultrasonic treatment appeared to have blurred morphology, but the majority of the bacteria still possessed intact cell membranes (Figure 4B–D). In contrast, more obvious disrupted membranes and altered cellular morphologies were observed in the *LMO* with SPDT treatment (Figure 4E), indicating that the SPDT treatment could effectively disrupt the bacteria membrane of *LMO*.

### 3.4. SPDT Induced Cytoplasmic Material Leakage in LMO

**Figure 5:**
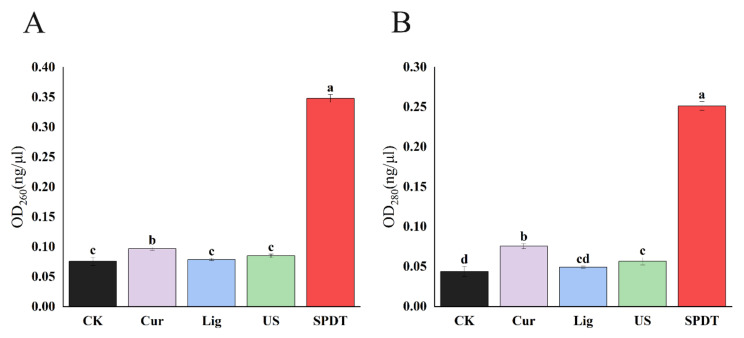
Release of 260 nm (**A**) and 280 nm (**B**) absorbing cytoplasmic materials from *Listeria monocytogenes.* CK: Control group without treatment; Cur: Curcumin-treated group; Lig: Light-illuminated group; US: Ultrasound-treated group; SPDT: Curcumin-mediated sono-photodynamic treated group. Different lowercase letters above the columns indicate the significant difference (*p* < 0.05).

As shown in Figure 5, the OD_260_ and OD_280_ of bacterial culture filtration were similar among the untreated bacteria and *LMO* in the control groups (Curcumin group; Light group; and Ultrasound group). In contrast, SPDT caused a significant increase in both the OD_260_ and OD_280_ of bacterial culture filtration, indicating SPDT could destroy the cell structure and cause the leakage of substances to the extracellular area.

### 3.5. SPDT Induced DNA Fragmentation and Protein Degradation in LMO

**Figure 6:**
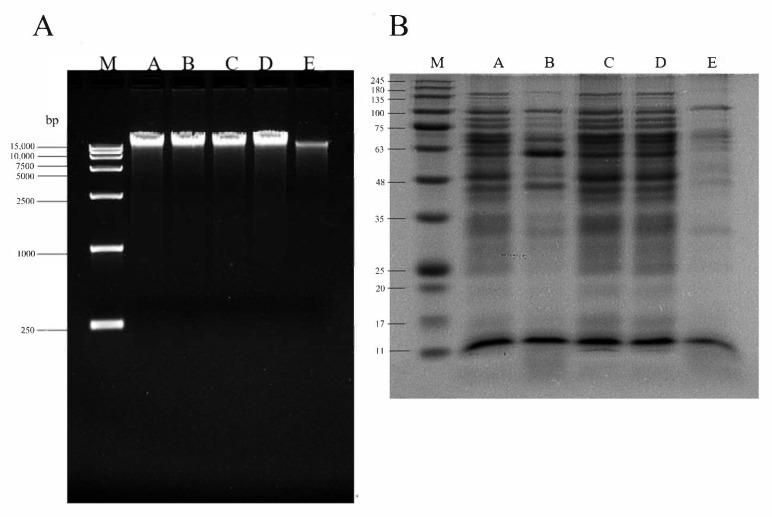
Curcumin-mediated sono-photodynamic treatment induced genomic DNA cleavage and general degradation of bacterial proteins in *Listeria monocytogenes*. (**A**) Agarose gel electrophoresis analysis of the cleavage of *Listeria monocytogenes* genomic DNA samples. (**B**) SDS-PAGE profile of *Listeria monocytogenes* total proteins. M: Marker; lane A: Control group without treatment; B: Curcumin-treated group; C: Light-illuminated group; D: Ultrasound-treated group; E: Curcumin-mediated sono-photodynamic treated group.

As shown in Figure 6, significant DNA degradation occurred in *LMO* upon SPDT treatment, as evidenced by the weak and smeared DNA bands being detected. Similarly, the protein bands in the SPDT-treated group also became scattered and blurred, with large proportions of the bands even disappearing. These phenomena also implied that SPDT triggered a universal protein degradation in *LMO*, making it impossible for the bacteria to metabolize properly, and thus leading to its death.

### 3.6. SPDT Induced Membrane Depolarization in LMO

**Figure 7:**
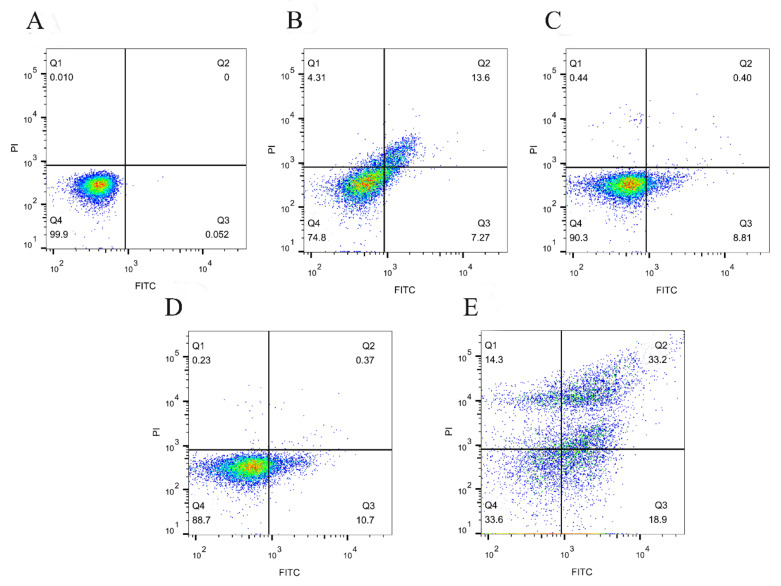
Membrane depolarization of *Listeria monocytogenes* detected by flow cytometry with Annexin V-FITC/PI double staining. (**A**) Control group without treatment; (**B**) Curcumin-treated group; (**C**) Light-illuminated group; (**D**) Ultrasound-treated group; (**E**) Curcumin-mediated sono-photodynamic treated group. The two-parameter dot plot divides the cells into four zones: Q1 indicating mechanically damaged cells (Annexin V-FITC-/PI+), Q2 indicating dead with membrane depolarization (Annexin V-FITC+/PI+), Q3 indicating bacteria with apoptosis-like event membrane depolarization (Annexin V-FITC+/PI−), and Q4 indicating live cells (Annexin V-FITC-/PI−).

As shown in Figure 7, the majority of untreated *LMO* showed the absence of annexin V-binding and propidium uptake (Q4, live status); while light illumination or ultrasound treatment slightly increased the percentage of bacterial cells being PI-negative and annexin V-positive. In contrast, bacterial cells in the SPDT group demonstrated massive outward exposure of phosphatidylserine, which is the biochemical hallmark of bacterial apoptosis-like events.

## 4. Discussion

The development of novel sterilization technologies that effectively deactivate bacteria with less impact on sensory and physical characteristics of foods is always a major focus in food science [33]. SPDT is a non-thermal sterilization technology that has already been used in clinical practice over the past few decades [34,35], exhibiting great potential in killing pathogenic bacteria. Recently, a number of studies have revealed that SPDT could effectively inactivate major food-borne pathogens (*E. coli*, *Salmonella*, and *S. aureus*) [26,36]. However, to the best of our knowledge, there is no report so far to suggest the efficacy of SPDT against *LMO*. Thus, in the current study, we assessed the effectiveness and mechanism of action of curcumin-mediated SPDT in killing *LMO*.

The obtained results demonstrated that the sterilization effect of SPDT was significantly enhanced with the extension of the sono-photodynamic processing time and increases in ultrasonic power and curcumin concentration. The optimal sterilization effect was achieved when the curcumin concentration was 50 μmol/L, the sono-photodynamic action time was 25 min, the ultrasonic power was 800 W and the blue LED light wavelength was 425 nm. The total number of *LMO* colonies under these parameters decreased by about 4.07 ± 0.15 log (equal to a bactericidal rate of 99.99 ± 0.01%), revealing a good bactericidal ability of the SPDT against *LMO*.

ROS production, which plays a crucial role in bactericidal activity, was investigated in the current study. Excessive production of ROS in bacteria can trigger bacterial death by multiple mechanisms, including damaging cellular components (e.g., bacterial lipids, DNA and proteins) [37], disrupting normal physiological metabolism [38], increasing permeability of cell membranes [38], even speeding up gene mutations [39], which ultimately lead to cell death [40]. Indeed, ROS generation was suggested as an important mechanism underlying the bactericidal activity of SPDT. For instance, hematoporphyrin monomethyl ether (HMME) and rose bengal (RB)-based nanoparticle-mediated SPDT was found to induce ROS generation with obvious bactericidal effects against methicillin-resistant *S. aureus* and extended-spectrum beta lactamase (ESBL)-producing *E. coli* [41]. Here, our results also suggested the SPDT induced excessive ROS in *LMO*, which led to cell membrane rupture and cell crumbling, as shown in the SEM images. Meanwhile, consistent with a number of studies [32,42], bacterial membrane damage can further result in the leakage of low-molecular-weight cytoplasmic constituents that have strong UV absorption at 260 nm and 280 nm. Indeed, these occurrences can also result in abnormal electrical potential across the membrane, thus affecting the physiological metabolism of the bacteria [43,44]. Meanwhile, as highly reactive molecules, ROS-induced large amounts of DNA damage and protein degradation were also observed in the current study. Indeed, ROS-induced oxidative damage to genomic DNA and proteins was also mentioned in previous literature [23,45,46].

Notably, previous studies also reported that DNA damage often led to bacterial apoptosis-like events, such as membrane depolarization [47]. Indeed, Maryam et al. also reported that curcumin-mediated sono-dynamic treatment effectively kills *S. mutans* via apoptosis-like death [47]. Here, our findings also showed SPDT resulted in the outward exposure of phosphatidylserine, suggesting that bacterial apoptosis-like events may also be involved in the killing effects of SPDT against *LMO*.

## 5. Conclusions

In previous studies, SPDT has demonstrated its desirable bactericidal activity against a number of food-borne bacteria (including *E. coli* [38], *S. aureus* [22,26] and *E. faecalis* [21]). Here, our study further demonstrated this non-thermal sterilization technique could effectively kill another important food-borne pathogen, *LMO*, which indicated that SPDT possessed a wide antimicrobial spectrum and could be a promising approach to efficiently lowered bacterial growth in foods. Admittedly, as a novel sterilization technology, further studies are needed before SPDT could become commercialized in the food industry. Taken together, curcumin activated by blue light and ultrasound is a potential non-thermal sterilization method for sono-photodynamic inactivation against *LMO*.

## Data Availability

Data is contained within the article.

## Supplementary Materials

The following are available online at https://www.mdpi.com/article/10.3390/foods11060808/s1, Figure S1: Representative plate pictures of LMO in CFU counting assay.

## References

[1] Pires S.M., Desta B.N., Mughini-Gras L., Mmbaga B.T., Fayemi O.E., Salvador E.M., Gobena T., Majowicz S.E., Hald T., Hoejskov P.S. (2021). Burden of foodborne diseases: Think global, act local. Curr. Opin. Food Sci..

[2] Giaouris E., Heir E., Desvaux M., Hébraud M., Møretrø T., Langsrud S., Doulgeraki A., Nychas G.J., Kačániová M., Czaczyk K. (2015). Intra- and inter-species interactions within biofilms of important foodborne bacterial pathogens. Front. Microbiol..

[3] Abebe E., Gugsa G., Ahmed M. (2020). Review on Major Food-Borne Zoonotic Bacterial Pathogens. J. Trop. Med..

[4] Su X., Cao G., Zhang J., Pan H., Zhang D., Kuang D., Yang X., Xu X., Shi X., Meng J. (2019). Characterization of internalin genes in *Listeria monocytogenes* from food and humans, and their association with the invasion of Caco-2 cells. Gut Pathog..

[5] Manso B., Melero B., Stessl B., Fernandez-Natal I., Jaime I., Hernandez M., Wagner M., Rovira J., Rodriguez-Lazaro D. (2019). Characterization of Virulence and Persistence Abilities of *Listeria monocytogenes* Strains Isolated from Food Processing Premises. J. Food Prot..

[6] Ghebrehiwet B., Jesty J., Peerschke E.I.B. (2002). gC1q-R/p33: Structure-Function Predictions from the Crystal Structure. Immunobiology.

[7] Zemser R.B., Martin S.E. (1998). Heat stability of virulence-associated enzymes from *Listeria monocytogenes* SLCC 5764. J. Food Prot..

[8] Schnupf P., Portnoy D.A. (2007). Listeriolysin O: A phagosome-specific lysin. Microbes Infect..

[9] Reddy S., Akgul A., Karsi A., Abdelhamed H., Wills R.W., Lawrence M.L. (2016). The role of *Listeria monocytogenes* cell wall surface anchor protein LapB in virulence, adherence, and intracellular replication. Microb. Pathog..

[10] Dinner S., Kaltschmidt J., Stump-Guthier C., Hetjens S., Ishikawa H., Tenenbaum T., Schroten H., Schwerk C. (2017). Mitogen-activated protein kinases are required for effective infection of human choroid plexus epithelial cells by *Listeria monocytogenes*. Microbes Infect..

[11] Drolia R., Tenguria S., Durkes A.C., Turner J.R., Bhunia A.K. (2018). *Listeria* Adhesion Protein Induces Intestinal Epithelial Barrier Dysfunction for Bacterial Translocation. Cell Host Microbe.

[12] Drolia R., Bhunia A.K. (2019). Crossing the Intestinal Barrier via *Listeria* Adhesion Protein and Internalin A. Trends Microbiol..

[13] Awuah G.B., Ramaswamy H.S., Economides A. (2007). Thermal processing and quality: Principles and overview. Chem. Eng. Process. Process Intensif..

[14] Misra N.N., Koubaa M., Roohinejad S., Juliano P., Alpas H., Inacio R.S., Saraiva J.A., Barba F.J. (2017). Landmarks in the historical development of twenty first century food processing technologies. Food Res. Int..

[15] Mtaoua H., Sánchez-Vega R., Ferchichi A., Martín-Belloso O. (2017). Impact of High-Intensity Pulsed Electric Fields or Thermal Treatment on the Quality Attributes of Date Juice through Storage. J. Food Process. Preserv..

[16] Zhang Z., Huang Z., Tong J., Wu Q., Pan Y., Malakar P.K., Zhao Y. (2021). An outlook for food sterilization technology: Targeting the outer membrane of foodborne gram-negative pathogenic bacteria. Curr. Opin. Food Sci..

[17] Pang X., Liu X., Cheng Y., Zhang C., Ren E., Liu C., Zhang Y., Zhu J., Chen X., Liu G. (2019). Sono-Immunotherapeutic Nanocapturer to Combat Multidrug-Resistant Bacterial Infections. Adv. Mater..

[18] McHale A.P., Callan J.F., Nomikou N., Fowley C., Callan B. (2016). Sonodynamic Therapy: Concept, Mechanism and Application to Cancer Treatment. Adv. Exp. Med. Biol..

[19] Akhtar F., Khan A.U., Misba L., Akhtar K., Ali A. (2021). Antimicrobial and antibiofilm photodynamic therapy against vancomycin resistant *Staphylococcus aureus* (VRSA) induced infection in vitro and in vivo. Eur. J. Pharm. Biopharm..

[20] Sadanala K.C., Chaturvedi P.K., Seo Y.M., Kim J.M., Jo Y.S., Lee Y.K., Ahn W.S. (2014). Sono-Photodynamic Combination Therapy: A Review on Sensitizers. Anticancer Res..

[21] Niavarzi S., Pourhajibagher M., Khedmat S., Ghabraei S., Chiniforush N., Bahador A. (2019). Effect of ultrasonic activation on the efficacy of antimicrobial photodynamic therapy: Evaluation of penetration depth of photosensitizer and elimination of *Enterococcus faecalis* biofilms. Photodiagnosis Photodyn. Ther..

[22] Drantantiyas N., Astuti S.D., Nasution A. Comparison microbial killing efficacy between sonodynamic therapy and photodynamic therapy. Proceedings of the International Seminar on Photonics, Optics, & Its Applications.

[23] Lai D., Zhou A., Tan B.K., Tang Y., Sarah Hamzah S., Zhang Z., Lin S., Hu J. (2021). Preparation and photodynamic bactericidal effects of curcumin-beta-cyclodextrin complex. Food Chem..

[24] Turrini E., Ferruzzi L., Fimognari C. (2014). Natural compounds to overcome cancer chemoresistance: Toxicological and clinical issues. Expert Opin. Drug Metab. Toxicol..

[25] Fan L., Idris Muhammad A., Bilyaminu Ismail B., Liu D. (2021). Sonodynamic antimicrobial chemotherapy: An emerging alternative strategy for microbial inactivation. Ultrason. Sonochem..

[26] Alves F., Gomes Guimaraes G., Mayumi Inada N., Pratavieira S., Salvador Bagnato V., Kurachi C. (2021). Strategies to Improve the Antimicrobial Efficacy of Photodynamic, Sonodynamic, and Sonophotodynamic Therapies. Lasers Surg. Med..

[27] Pourhajibagher M., Bahador A. (2021). Attenuation of *Aggregatibacter actinomycetemcomitans* virulence using curcumin-decorated nanophytosomes-mediated photo-sonoantimicrobial chemotherapy. Sci. Rep..

[28] Bhavya M.L., Hebbar H.U. (2019). Sono-photodynamic inactivation of *Escherichia coli* and *Staphylococcus aureus* in orange juice. Ultrason. Sonochem..

[29] Wang D., Zhou F., Lai D., Zhang Y., Hu J., Lin S. (2021). Curcumin-mediated sono/photodynamic treatment preserved the quality of shrimp surimi and influenced its microbial community changes during refrigerated storage. Ultrason. Sonochem..

[30] Hu J., Lin S., Tan B.K., Hamzah S.S., Lin Y., Kong Z., Zhang Y., Zheng B., Zeng S. (2018). Photodynamic inactivation of *Burkholderia cepacia* by curcumin in combination with EDTA. Food Res. Int..

[31] Su H.-L., Chou C.-C., Hung D.-J., Lin S.-H., Pao I.C., Lin J.-H., Huang F.-L., Dong R.-X., Lin J.-J. (2009). The disruption of bacterial membrane integrity through ROS generation induced by nanohybrids of silver and clay. Biomaterials.

[32] Yao C., Li X., Bi W., Jiang C. (2014). Relationship between membrane damage, leakage of intracellular compounds, and inactivation of *Escherichia coli* treated by pressurized CO_2_. J. Basic Microbiol..

[33] Morales-de la Peña M., Welti-Chanes J., Martín-Belloso O. (2019). Novel technologies to improve food safety and quality. Curr. Opin. Food Sci..

[34] Wang X., Jia Y., Wang P., Liu Q., Zheng H. (2017). Current status and future perspectives of sonodynamic therapy in glioma treatment. Ultrason. Sonochem..

[35] Hirschberg H., Madsen S.J. (2017). Synergistic efficacy of ultrasound, sonosensitizers and chemotherapy: A review. Ther. Deliv..

[36] Nakonechny F., Nisnevitch M., Nitzan Y., Nisnevitch M. (2013). Sonodynamic excitation of Rose Bengal for eradication of gram-positive and gram-negative bacteria. Biomed. Res. Int..

[37] Lichtenberg D., Pinchuk I. (2015). Oxidative stress, the term and the concept. Biochem. Biophys. Res. Commun..

[38] Wu M., Zhang Z., Liu Z., Zhang J., Zhang Y., Ding Y., Huang T., Xiang D., Wang Z., Dai Y. (2021). Piezoelectric nanocomposites for sonodynamic bacterial elimination and wound healing. Nano Today.

[39] Lushchak V.I., Storey K.B. (2021). Oxidative stress concept updated: Definitions, classifications, and regulatory pathways implicated. EXCLI J..

[40] Ezraty B., Gennaris A., Barras F., Collet J.F. (2017). Oxidative stress, protein damage and repair in bacteria. Nat. Rev. Microbiol..

[41] Xu F., Hu M., Liu C., Choi S.K. (2017). Yolk-structured multifunctional up-conversion nanoparticles for synergistic photodynamic-sonodynamic antibacterial resistance therapy. Biomater. Sci..

[42] Li X.-F., Feng X.-Q., Yang S., Fu G.-Q., Wang T.-P., Su Z.-X. (2010). Chitosan kills *Escherichia coli* through damage to be of cell membrane mechanism. Carbohydr. Polym..

[43] Costley D., Nesbitt H., Ternan N., Dooley J., Huang Y.Y., Hamblin M.R., McHale A.P., Callan J.F. (2017). Sonodynamic inactivation of Gram-positive and Gram-negative bacteria using a Rose Bengal-antimicrobial peptide conjugate. Int. J. Antimicrob. Agents.

[44] Alves F., Ayala E.T.P., Pratavieira S. (2021). Sonophotodynamic Inactivation: The power of light and ultrasound in the battle against microorganisms. J. Photochem. Photobiol..

[45] Ray S.K., Dhakal D., Hur J., Lee S.W. (2020). Visible light driven MoS2/alpha-NiMoO4 ultra-thin nanoneedle composite for efficient *Staphylococcus aureus* inactivation. J. Hazard. Mater..

[46] Wu J., Mou H., Xue C., Leung A.W., Xu C., Tang Q.J. (2016). Photodynamic effect of curcumin on Vibrio parahaemolyticus. Photodiagnosis Photodyn. Ther..

[47] Pourhajibagher M., Rahimi Esboei B., Hodjat M., Bahador A. (2020). Sonodynamic excitation of nanomicelle curcumin for eradication of *Streptococcus mutans* under sonodynamic antimicrobial chemotherapy: Enhanced anti-caries activity of nanomicelle curcumin. Photodiagn. Photodyn. Ther..

