# Supplementary figures and images for: Curcumin-Mediated Sono-Photodynamic Treatment Inactivates Listeria monocytogenes via ROS-Induced Physical Disruption and Oxidative Damage
Source: Foods. 2022 Mar 11;11(6):808. doi: 10.3390/foods11060808 (PMC8947418; doi:10.3390/foods11060808)

**Supplementary Figure S1**

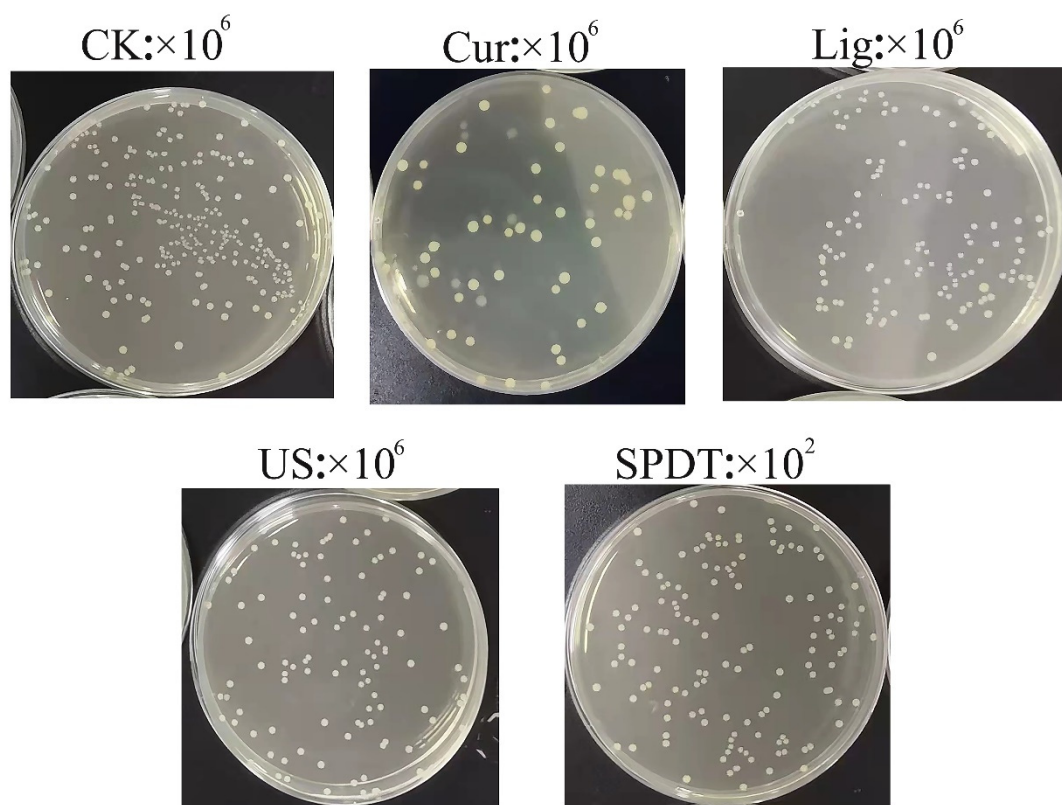

**Representative plate pictures of LMO in CFU counting assay.**

Supplement: Supplementary file 1 [file foods-11-00808-s001.zip › foods-1614864-supplementary.pdf]
